# 11β-HSD1 suppresses cardiac fibroblast CXCL2, CXCL5 and neutrophil recruitment to the heart post MI

**DOI:** 10.1530/JOE-16-0501

**Published:** 2017-04-11

**Authors:** Katie J Mylonas, Neil A Turner, Sumia A Bageghni, Christopher J Kenyon, Christopher I White, Kieran McGregor, Robert A Kimmitt, Richard Sulston, Valerie Kelly, Brian R Walker, Karen E Porter, Karen E Chapman, Gillian A Gray

**Affiliations:** 1 University/BHF Centre for Cardiovascular ScienceUniversity of Edinburgh, Queen’s Medical Research Institute, Edinburgh, UK; 2 Division of Cardiovascular & Diabetes ResearchLeeds Institute of Cardiovascular & Metabolic Medicine (LICAMM), School of Medicine, University of Leeds, Leeds, UK

**Keywords:** heart, myocardial infarction, neutrophil, fibroblast, chemokine, GDF-15

## Abstract

We have previously demonstrated that neutrophil recruitment to the heart following myocardial infarction (MI) is enhanced in mice lacking 11β-hydroxysteroid dehydrogenase type 1 (11β-HSD1) that regenerates active glucocorticoid within cells from intrinsically inert metabolites. The present study aimed to identify the mechanism of regulation. In a mouse model of MI, neutrophil mobilization to blood and recruitment to the heart were higher in 11β-HSD1-deficient (*Hsd11b1^−^^/^^−^*) relative to wild-type (WT) mice, despite similar initial injury and circulating glucocorticoid. In bone marrow chimeric mice, neutrophil mobilization was increased when 11β-HSD1 was absent from host cells, but not when absent from donor bone marrow-derived cells. Consistent with a role for 11β-HSD1 in ‘host’ myocardium, gene expression of a subset of neutrophil chemoattractants, including the chemokines *Cxcl2* and *Cxcl5*, was selectively increased in the myocardium of *Hsd11b1^−^^/^^−^* mice relative to WT. SM22α-Cre directed disruption of *Hsd11b1* in smooth muscle and cardiomyocytes had no effect on neutrophil recruitment. Expression of *Cxcl2* and *Cxcl5* was elevated in fibroblast fractions isolated from hearts of *Hsd11b1^−^*^/^*^−^* mice post MI and provision of either corticosterone or of the 11β-HSD1 substrate, 11-dehydrocorticosterone, to cultured murine cardiac fibroblasts suppressed IL-1α-induced expression of *Cxcl2* and *Cxcl5*. These data identify suppression of CXCL2 and CXCL5 chemoattractant expression by 11β-HSD1 as a novel mechanism with potential for regulation of neutrophil recruitment to the injured myocardium, and cardiac fibroblasts as a key site for intracellular glucocorticoid regeneration during acute inflammation following myocardial injury.

## Introduction

Ischemic cell death associated with myocardial infarction (MI) prompts the recruitment and activation of immune cells to ensure repair (Epelman & Mann 2012, Frangogiannis 2012). Neutrophils are recruited early, removing necrotic tissue and matrix debris from the damaged myocardium (Frangogiannis 2012, Yan *et al*. 2013), but they are also important to ensure transition to repair. Macrophage polarization toward a reparative phenotype in the healing myocardium is promoted by neutrophil-derived gelatinase-associated lipocalin and by efferocytosis of apoptotic neutrophils (Chen *et al*. 2014, Horckmans *et al*. 2017). However, neutrophil recruitment requires tight regulation to minimize the risk of tissue damage due to release of pro-inflammatory mediators, matrix metalloproteinases and oxygen free radicals (Jordan *et al*. 1999, Ma *et al*. 2013).

Systemic glucocorticoid concentration is increased by the activation of the hypothalamic–pituitary–adrenal axis early after MI (Morrison *et al*. 1976). Active glucocorticoid (predominantly cortisol in man and corticosterone in rats and mice) is also regenerated within cells from intrinsically inert metabolites (cortisone and 11-dehydrocorticosterone 11-DHC) by the intracellular enzyme 11β-hydroxysteroid dehydrogenase type 1 (11β-HSD1). Cell-specific enhancement of 11βHSD1 availability by IL-1 (Tomlinson *et al*. 2001) and by glucocorticoid (Chapman *et al*. 2006) serves to locally amplify glucocorticoid action during inflammation (Sun & Myatt 2003, Chapman *et al*. 2013*a*). When 11β-HSD1 is deficient or inhibited, neutrophil-driven inflammation is increased in experimental sterile peritonitis (Gilmour *et al*. 2006), arthritis (Coutinho *et al*. 2012, Nanus *et al*. 2015) and carrageenan-induced pleurisy (Chapman *et al*. 2006, Coutinho *et al*. 2012). In an earlier study, we found that early neutrophil recruitment to the injured myocardium also increases in 11β-HSD1-deficient mice following induction of MI by coronary artery ligation, and this is associated with increased pro-reparative macrophage polarization and reduced detrimental remodeling (McSweeney *et al*. 2010). This suggests a key role for intracellular corticosteroid regeneration in regulating neutrophil recruitment to the injured heart, but the underlying mechanisms are unknown (Gray *et al*. 2017).

Neutrophil recruitment requires mobilization from the bone marrow to the blood in response to chemoattractant signals from injured tissue, as well as adhesion to endothelial cells and migration into the tissue. Previous studies have focused on the latter, showing that neutrophil 11β-HSD1 activity is increased during inflammation and that this activity regulates the expression of L-selectin, CD11b and annexin (Kardon *et al*. 2008, Coutinho *et al*. 2016). However, the heart releases chemoattractants that include interleukin (IL)-1 and the CXCR2-activating ELR chemokines such as CXCL1 (KC), CXCL2 (MIP-2α; macrophage inflammatory protein 2-alpha) and CXCL5 (LIX), that are essential for neutrophil recruitment after injury (Chandrasekar *et al*. 2001, Kobayashi 2008, Frangogiannis 2012). Recent studies have also identified growth-differentiation factor-15 (GDF-15) as a novel inhibitor of neutrophil recruitment post MI (Kempf *et al*. 2011). Regulation of the expression of these key molecules is a potential additional mechanism through which glucocorticoid, generated in myocardial cells by 11β-HSD1, suppresses neutrophil recruitment after injury.

11β-HSD1 is expressed by a number of cells found in the heart with the potential to release chemoattractants following injury, including cardiomyocytes (Mazancova *et al*. 2005), vascular smooth muscle (Hatakeyama *et al*. 2001), mast cells (Coutinho *et al*. 2013) and resident macrophages (Gilmour *et al*. 2006) and fibroblasts (Sun & Myatt 2003). In a recent study (White *et al*. 2016), we reported that 11β-HSD1 deletion in cardiac and vascular smooth muscle has no effect on chronic remodeling post MI, suggesting that these cells are not likely to be a key site for regulation of myocardial inflammation and repair.

The present study aimed to investigate whether changes in myocardial neutrophil content are accompanied by changes in neutrophil mobilization from bone marrow to the blood; whether myocardial neutrophil chemoattractant expression post MI is influenced by 11β-HSD1 availability and key cellular sites of 11β-HSD1 expression that determine neutrophil recruitment.

## Materials and methods

### Mice

Experiments used adult male mice with global deficiency on a C57BL/6 genetic background (*Hsd11b1*^−/−^) (Kipari *et al*. 2013) (Supplementary Fig. 1, see section on supplementary data given at the end of this article), with WT C57BL/6 mice used as controls. Mice with deletion targeted to vascular smooth muscle and cardiomyocytes (White *et al*. 2016)*(Hsd11b1^fl/fl^Sm22α-Cre^+^; Hsd11b1*^CVCre+^) were generated by crossing *Sm22α-Cre* mice with *Hsd11b1^fl/fl^* mice, homozygous for a ‘floxed’ allele of *Hsd11b1* (generated by Artemis Pharmaceuticals, Cologne, Germany), directly onto a C57BL/6J background. LoxP sites flanked exon 3 of the mouse *Hsd11b1* gene. Excision of this exon results in a ‘null allele’ by ‘out-of-frame splicing’ from exon 2 to exon 4. Controls were *Hsd11b1^fl/fl^* (*Cre−; Hsd11b1*^CVCre−^) littermates.

Male mice (6–14 weeks old) were bred and maintained in conventional barrier unit facilities at the University of Edinburgh. Experimental and control mice, WT or Cre− littermates (*Hsd11b1^CVCre^*^−^), were age matched. All animal work was compliant with IACUC guidelines, conducted in accordance with the UK Government Animals (Scientific Procedures) Act 1986 and was approved by the University of Edinburgh Animal Welfare and Ethical Review Board.

### Generation of chimeric mice

Chimeric mice were created by exposing WT and *Hsd11b1*^−/−^ mice to a single lethal dose of 10.5 Gy radiation. This was followed by i.v. injection of 1 × 10^7^ donor bone marrow cells harvested from the femurs and tibias of WT or *Hsd11b1*^−/−^ mice. To control for the effects of irradiation, ‘irradiation control’ animals were generated wherein WT recipients received WT bone marrow. In ‘host KO’ animals, *Hsd11b1*^−/−^ recipients received WT donor cells, so that all quickly dividing cells prone to radiation, including bone marrow leucocytes, were replaced by cells with a WT phenotype, but host cells were *Hsd11b1* deficient. In ‘BMKO’ animals, WT recipients received *Hsd11b1*^−/−^ bone marrow, resulting in *Hsd11b1*^−/−^ neutrophils and WT radio-resistant host tissue. Chimeric animals were housed under pathogen-free conditions in individually ventilated cages and given Baytril antibiotic (2.5%) in the drinking water for 1 week before and 4 weeks after bone marrow transplantation. Chimeras were allowed to recover for 8 weeks before appropriate reconstitution was confirmed by flow cytometry of tail vein blood and CAL surgery was performed.

### Coronary artery ligation (CAL) surgery

CAL surgery for induction of myocardial infarction was carried out as we have previously described (White *et al*. 2016). Mice were anesthetized with isoflurane (2%), which was maintained for the length of the procedure, and received appropriate analgesia (buprenorphine 0.05 mg/kg s.c. before surgery and 24 h later). The trachea was intubated and the lungs were ventilated mechanically at 120 strokes/min. The left thorax was opened at the fourth intercostal space, before the left descending coronary artery ligated with a 6.0-prolene suture. After closing the thorax, animals received oxygen in the absence of anesthesia until fully conscious.

### Plasma troponin measurement

Tail vein blood was collected from mice 24 h post CAL surgery, following administration of analgesic (buprenorphine 0.05 mg/kg s.c.), into 10 mM sodium citrate buffer and centrifuged at 2400 ***g*** for 10 min at 4°C to produce a plasma phase. Cardiac Troponin I (Tn-I) was measured in the plasma using the mouse high-sensitivity Tn-I ELISA kit according to the manufacturers instructions (Life Diagnostics, Staffordshire, UK).

### Tissue collection and immunohistochemistry

Mice were terminally anesthetized with saline containing ketamine at 50 mg/kg and medetomidine at 0.5 mg/kg by i.p. injection. Hearts were perfusion fixed by infusing heparinized saline (10 IU/mL heparin), and then 10% formalin at physiological pressure (100 mmHg) via the abdominal aorta. Hearts were placed in 10% formalin overnight before processing to wax and paraffin embedding for GR1+ immunohistochemistry (IHC; antibody used in Table 1) as described previously (McSweeney *et al*. 2010). For quantification, sections were tiled at ×40 magnification (Image Pro6.2, Stereologer Analyser 6 MediaCybernetics). The % area stained with the GR1 antibody (dark brown) was calculated within the infarct and border area.

**Table 1:** Antibodies.

**Name and clone**	**Conjugate**	**Dilution**	**Supplier and cat #**
Primary antibodies			
Rat anti-mouse Gr1 (Ly6G and Ly6C)	None	1:100	BD Pharmingen #550291
Clone RB6-8C5			
Mouse anti-mouse CD45	PE Cy7	1:100	Biolegend #109830
Clone 105			
Rat anti-mouse CD11b	Alexafluor 700	1:100	Biolegend #101222
Clone M1/70			
Rat anti-mouse Ly6G	Pacific Blue	1:100	Biolegend #127612
Clone 1A8			
Rat anti-mouse CXCR4	Alexafluor 647	1:100	Biolegend #129201
Clone TG12/CXCR4			
Anti-mouse CXCR2	PerCp cy5.5	1:100	Biolegend #129103
Clone TG11/CXCR2			
Sheep anti-mouse 11β-HSD1	None	1:100	In house
Secondary antibodies			
Rabbit anti-rat IgG	Biotin	1:200	Vector # BA4001
Donkey anti-sheep	Alexa Fluor 488	1:100	Abcam # ab150177

### Corticosterone radioimmunoassay

Plasma corticosterone at the diurnal nadir, one day post surgery, was measured by radioimmunoassay as described previously (Kotelevtsev *et al*. 1997).

### Flow cytometry of blood and bone marrow cells

Mice were killed by cervical dislocation, and bone marrow cells were harvested by flushing the femur and tibia with phosphate buffered saline (PBS; Thermo Fisher) as described previously (Kipari *et al*. 2013). Erythrocytes were lysed (red blood cell lysis buffer; Sigma-Aldrich). 0.5 × 10^6^ singly-suspended cells were then blocked with 10% mouse serum for 20 min on ice, and then incubated for 30 min on ice with appropriate dilutions of antibodies of interest (Table 1) in PBS containing 10% mouse serum. Blood was collected in 10 mM sodium citrate buffer and antibodies were added directly to the blood. Intracellular staining of 11β-HSD1 in blood neutrophils was carried out as described previously (De Sousa Peixoto *et al*. 2008). Cells were fixed and permeabilized using a kit (Fix and Perm, Invitrogen; Thermo Fisher) to facilitate intracellular immunostaining of 11βHSD1. Cells were then washed in PBS before acquisition and analysis (BD FACS LSR Fortessa and FlowJo software; Oregon, USA). The gating strategy for analyzing blood neutrophils is shown in Supplementary Fig. 2.

### Fibroblast isolation post MI

Mice were terminally anesthetized with saline containing ketamine at 50 mg/kg and medetomidine at 0.5 mg/kg by i.p. injection. Heart tissue was digested before fibroblast isolation. Briefly, infarct and surrounding border heart tissues were chopped into small pieces and digested in collagenase D and DNase 1 (2.5 mg/mL collagenase D; 60 U/mL DNAse 1; Ambion) in HBSS (GIBCO; Thermo Fisher) at 37°C for 30 min following dissociation by gentleMacs Dissociator (according to manufacturer’s instructions; Miltenyi; Surrey, UK). The digested tissue was gently disaggregated and filtered through a 30 µm cell strainer to remove larger cells (including cardiomyocyes). Cells were then centrifuged at 300 ***g*** for 5 min and washed in PBS. Cardiac fibroblasts were isolated magnetically using a Miltenyi neonatal cardiac fibroblast isolation kit (*MACS*), according to manufacturer’s instructions.

Three cellular fractions were produced from this isolation method (Fractions 1–3). These were characterized by qPCR (Supplementary Fig. 4) and flow cytometry using specific cellular markers. Flow cytometry analysis revealed that Fraction 1 contained the highest levels of CD45 and platelet endothelial cell adhesion molecule (Pecam1; CD31; data not shown) suggesting enrichment for leukocytes (Nakano *et al*. 1990) and endothelial cells, respectively (Newman 1997), and this was confirmed by qPCR analysis (Supplementary Fig. 4). Fraction 2 highly expressed the fibroblast markers Discoidin domain receptor 2 (*Ddr2*; Supplementary Fig. 4) and type 1 collagen A1 (*Col1a1*; Supplementary Fig. 4), suggesting that it was enriched for fibroblasts. Fraction 3, which highly expressed Thy1 cell surface antigen (*Thy-1*; CD90), was also positive for the cardiac fibroblast markers *Ddr2* and *Col1a1* (Supplementary Fig. 4). In the heart, DDR2 is the most specific marker of cardiac fibroblasts (Camelliti *et al*. 2005). Thy1 is only expressed by a subset of fibroblasts (Willis *et al*. 1994). All isolated *Ddr2* and *Col1a1* (*Ddr2+Col1a1+*)-expressing cells (Fractions 2; *Thy1^l^*^ow^ and 3; *Thy1*^high^) were considered enriched for fibroblasts, and Fraction 1 enriched for leukocytes and endothelial cells. These cellular fractions from WT and *Hsd11b1*^−/−^ mice were analyzed by qPCR for the production of various genes of interest (Table 2). qPCR data were also analyzed on fractions enriched for both *Thy1*^low^ and T*hy1*^high^ fibroblasts from WT mice alone post MI (Supplementary Fig. 5).

**Table 2:** Applied Biosystems gene expression arrays used.

Gene name	TAQman gene expression array
Glyceraldehyde 3-phosphate dehydrogenase (Gapdh)	Mm99999915_gl
CXCL1 (*Cxcl1*)	Mm004207460_m1
NLRP3 (*Nlrp3*)	Mm04210225_m1
Chemokine (C-C motif) ligand 3 (Ccl3)	Mm00441259_g1
L-selectin	Mm00441291_m1
CXCL5 (*Cxcl5*)	Mm004 36451_g1
Interleukin (IL-)1β (*Il1b*)	Mm00434228_m1
IL-6 (*Il6*)	Mm00446190_m1
CXCL2 (*Cxcl2*)	Mm00436450_m1
Growth differentiation factor 15 (*Gdf15*)	Mm00442228_m1
CXCL12 (*Cxcl12*)	Mm00445553_m1
11βHSD1 (*Hsd11b1*)	Mm 00476182_m1
Thy1 (*Yhy1*)	Mm00493682_g1
CD31 (*Pecam1*)	Mm01242584_m1
DDR2 (*Ddr2*)	Mm00445615_m1
Col1a1 (*Col1a1*)	Mm00801666_g1

### Cardiac fibroblast culture

Mice were killed by exposure to increasing concentrations of CO_2_. Cardiac fibroblasts were collected from hearts by collagenase digestion and cultured as described previously for human cells (Turner *et al*. 2003). Briefly, ventricular tissue from 6- to 8-week-old mice was washed in PBS, and then minced with scissors and digested by adding Worthington Type II collagenase (600 IU/mL) for 90 min with occasional shaking at 37°C. After centrifugation of the suspension, the cells were washed twice in DMEM cell culture medium, before seeding cells into a T25 tissue culture flask. Non-adherent cells were removed after 30 min, and the remaining cells were incubated with full growth medium (DMEM + 10% FCS; Thermo Fisher) overnight. The next day, cells were washed twice with PBS to remove any residual blood cells before the addition of fresh growth medium. For experiments, cells at passages 1–2 were plated into 6-well plates and serum-starved overnight before incubation for 24 h with medium containing 1 ng/mL IL-1α together with 200 nM corticosterone, 200 nM 11-dehydrocoticosterone (11-DHC; inert form) or ethanol (vehicle control). Conditioned media were collected for ELISA analysis. RNA was extracted from cell pellets and cDNA synthesized for qPCR as described (Turner *et al*. 2007) (TaqMan gene expression arrays; Table 2).

### Chemokine quantification from fibroblast culture supernatant

ELISAs for CXCL2 and CXCL5 in conditioned media from fibroblast cultures were carried out according to manufacturer’s instructions (R&D Systems).

### RNA extraction and quantitative PCR (qPCR)

RNA was extracted from bone marrow, cardiac left ventricular/infarct tissue or fibroblast cell fractions using the TRIzol method (according to manufacturers instructions; Thermo Fisher), and this was then reverse transcribed to cDNA (Applied Biosystems high-capacity cDNA reverse transcription kit or Promega reverse transcription system). TaqMan gene expression arrays (Applied Biosystems) were used to quantify mRNA expression of genes (Table 2). Results were normalized for GAPDH expression and presented as fold increases over average control level analyzed in parallel, unless otherwise stated.

### Statistical analyses

All values are expressed as mean ± s.e.m. Unpaired Student’s *t*-test or ANOVA with Newman–Keuls post-hoc test were used for analysis. *P* values <0.05 denote statistical significance, *P < 0.05, **P < 0.01, ***P < 0.005.

## Results

### Global deletion of *Hsd11b1* enhances neutrophil mobilization to blood and recruitment to the heart after MI, without changing infarct injury or plasma corticosterone concentration

**Figure 1:**
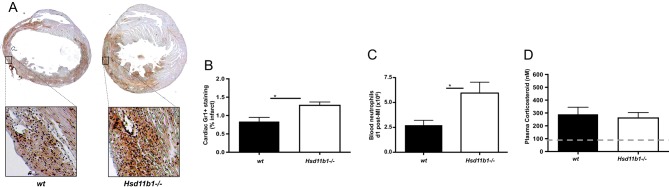
Increased neutrophil mobilization and recruitment to the heart in *Hsd11b1^−^^/^^−^* mice post MI. (A) Representative images of Gr-1 IHC of day 2 infarcted hearts from both WT (left) and *Hsd11b1^−^^/^^−^* mice (right). (B) Analysis of the % Gr-1+ immunostaining in infarct and border regions of heart sections from *Hsd11b1^−^^/^^−^* vs WT animals (*n* = 7/5). (C) Neutrophil numbers in the blood of WT vs *Hsd11b1^−^^/^^−^* mice measured by flow cytometry at 1 day post MI (*n* = 4/3). (D) Plasma corticosteroid levels were raised post MI and did not differ between WT and *Hsd11b1^−^^/^^−^* mice (*n* = 7/6; dashed line represents level for naïve C57BL/6 mice, approx. 50–100 ng/mL). *P < 0.05.

Deletion of 11β-HSD1 in the hearts of *Hsd11b1^−^^/^^−^* mice was confirmed by qPCR (Supplementary Fig. 1A) and Western blot (Supplementary Fig. 1B). After induction of MI, initial cardiac damage was similar in WT and *Hsd11b1^−^^/^^−^* mice as measured by the concentration of troponin I in tail blood collected 24 h post injury (Supplementary Fig. 1C). Analysis of hearts collected from these mice showed that Gr-1 immunoreactive neutrophil content in the infarct and border of *Hsd11b1^−^^/^^−^* mice was significantly higher than that of WT (Fig. 1A and B; *P* < 0.05), confirming our previous observations (McSweeney *et al*. 2010). Flow cytometry revealed that blood from *Hsd11b1^−^^/^^−^* mice also contained significantly more neutrophils (CD45+CD11b+Ly6G+cells; Supplementary Fig. 2) post MI compared to WT (Fig. 1C, *P*  < 0.05). Plasma corticosterone concentration in morning blood samples was increased to similar levels in WT and *Hsd11b1^−^^/^^−^* mice 24 h after induction of MI (Fig. 1D), excluding this as a contributing factor to alteration in neutrophil mobilization or recruitment.

### Increased neutrophil mobilization to blood is associated with reduced neutrophil expression of CXCR4, but not CXCR2, on bone marrow and blood neutrophils

To investigate whether increased mobilization of neutrophils to the blood of *Hsd11b1^−^^/^^−^* mice could be explained by changes in the expression of receptors determining retention or mobilization in the bone marrow, the surface expression of CXCR2 and CXCR4 on neutrophils was determined by flow cytometry. The surface expression of CXCR4, a receptor involved in bone marrow retention of neutrophils (Eash *et al*. 2010), was found to be reduced on bone marrow neutrophils collected from *Hsd11b1^−^^/^^−^*mice after MI (Supplementary Fig. 3A; *P* < 0.05), but there was no change in surface expression of the receptor determining mobilization, CXCR2 (Supplementary Fig. 3C). Gene expression of *Cxcl12* (SDF-1), the primary ligand for CXCR4, was not modified in the bone marrow of *Hsd11b1^−^^/^^−^* compared with WT (Supplementary Fig. 3B).

### Increased neutrophil mobilization to blood depends on the absence of *Hsd11b1* gene expression in host, not donor bone marrow-derived, cells in bone marrow chimeric mice

**Figure 2:**
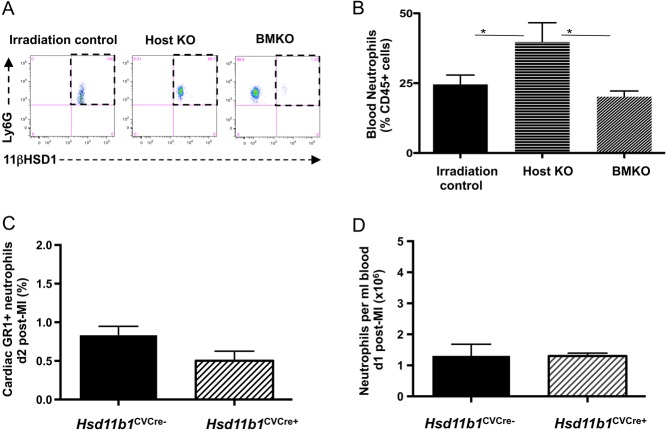
Absence of gene expression in host cells in bone marrow chimera causes increased neutrophil mobilization but targeted deletion of *Hsd11b1^−^^/^^−^* in smooth muscle cells and cardiomyocytes does not affect neutrophil mobilization or recruitment post MI. Flow cytometric analysis confirms appropriate presence or absence of 11β-HSD1 in the neutrophils of chimeric mice (A). Blood neutrophil numbers were measured in chimeric mice (B; *n* = 7/6/6, *P* < 0.05). (C) Neutrophils present in the heart 2 days post MI in control (*Hsd11b1*^CVCre−^) vs experimental animals (*Hsd11b1*^CVCre+^). (D) Analysis of blood neutrophil numbers in control (*Hsd11b1*^CVCre−^) vs experimental animals (*Hsd11b1*^CVCre+^) at 1 day post MI. *n* = 4/4.

To investigate whether increased mobilization of neutrophils to the blood results from direct effects of *Hsd11b1* deletion in neutrophils, bone marrow chimeric mice with *Hsd11b1* deletion in either donor bone marrow cells (BMKO) or in host cells (Host KO) were prepared. For irradiation controls, WT bone marrow was transferred into irradiated WT hosts. Flow cytometric analysis of intracellular 11β-HSD1 protein in blood neutrophils confirmed appropriate removal. Irradiation control and Host KO neutrophils were positive for 11β-HSD1 expression, but BMKO neutrophils did not express this protein (dashed box; Fig. 2A). In these bone marrow chimeric mice, neutrophil mobilization to the blood was increased relative to irradiation control mice in host KO animals post MI (Fig. 2B; *P* < 0.05), but not in BMKO mice (Fig. 2B). These data suggest that it is *Hsd11b1* in a component of the host tissue, rather than in bone marrow-derived cells, that determines increased mobilization of neutrophils to the blood following MI.

### 11β-HSD1 deletion in cardiomyocytes and vascular smooth muscle cells does not influence neutrophil mobilization or accumulation in the myocardium

Cardiomyocytes and vascular smooth muscle are key sites of 11β-HSD1 expression in the heart. To investigate whether 11β-HSD1 activity here might account for enhanced neutrophil mobilization to the blood and recruitment to the heart, MI was induced in mice with targeted deletion of *Hsd11b1* in smooth muscle and cardiomyocytes (White *et al*. 2016). However, floxed control, *Hsd11b1*^CVCre−^ and *Hsd11b1*^CVCre+^ mice all had similar numbers of neutrophils in the heart (Fig. 2C) and blood (Fig. 2D) following coronary artery ligation.

### 11β-HSD1 deletion increases cardiac expression of a subset of neutrophil chemoattractant genes after MI

**Figure 3:**
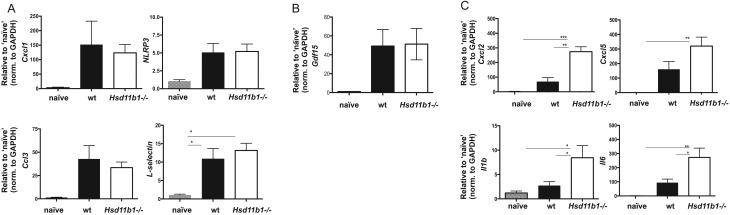
Inflammatory cytokines and chemokines involved in neutrophil recruitment increase in the heart post MI. qPCR was carried out on infarct and border cardiac tissue from WT or *Hsd11b1^−^^/^^−^* mice 24 h post MI and compared to naïve WT ventricular tissue. (A) *Cxcl1*, *NLRP2*, *Ccl3* and the adhesion molecule, *L-selectin*, increased in infarcted tissue compared to naïve myocardium but did not differ between WT and *Hsd11b1^−^^/^^−^* infarcts. (B) Expression of the neutrophil inhibitory peptide *Gdf-15* was not modified in hearts from *Hsd11b1^−^^/^^−^* mice. (C) *Cxcl2*, *Cxcl5*, *Il1b* and *Il6* were all expressed to a greater extent in *Hsd11b1^−^^/^^−^* than in WT infarct tissue. (C) *P < 0.05, **P < 0.01, ***P < 0.005. In representative experiments shown, *n* = 4/3, norm., normalized to GAPDH.

To investigate potential mediators of increased neutrophil recruitment to the heart, expression of a panel of genes encoding molecules known to regulate neutrophil recruitment was assessed in the left ventricle of hearts from *Hsd11b1*^−/−^ and WT mice. Cardiac expression levels of genes encoding the neutrophil chemoattractants *Cxcl1* (KC) and *Ccl3* (MIP-1α), as well as the inflammasome *Nlrp3*, and the adhesion molecule, *L-selectin* were all increased in hearts collected after MI relative to control hearts, but expression of these genes was not modified in hearts from *Hsd11b1*^−/−^ mice relative to WT (Fig. 3A; *P* < 0.05). Expression of the neutrophil inhibitory peptide *Gdf-15* was also increased post MI, consistent with previous observations (Kempf *et al*. 2011), but was not modified in hearts from *Hsd11b1*^−/−^ mice relative to WT (Fig. 3B). In contrast, expression of genes encoding *Cxcl2* (MIP-2α), *Cxcl5* (LIX), *Il1b* (IL-1β) and *Il6* (IL-6) was increased in the heart after MI and further increased in hearts from *Hsd11b1*^−^*^/^*^−^ mice compared to WT mice (Fig. 3C).

### *Cxcl2* and *Cxcl5* expression is increased in fibroblasts isolated from infarcted hearts of mice deficient in 11β-HSD1

**Figure 4:**
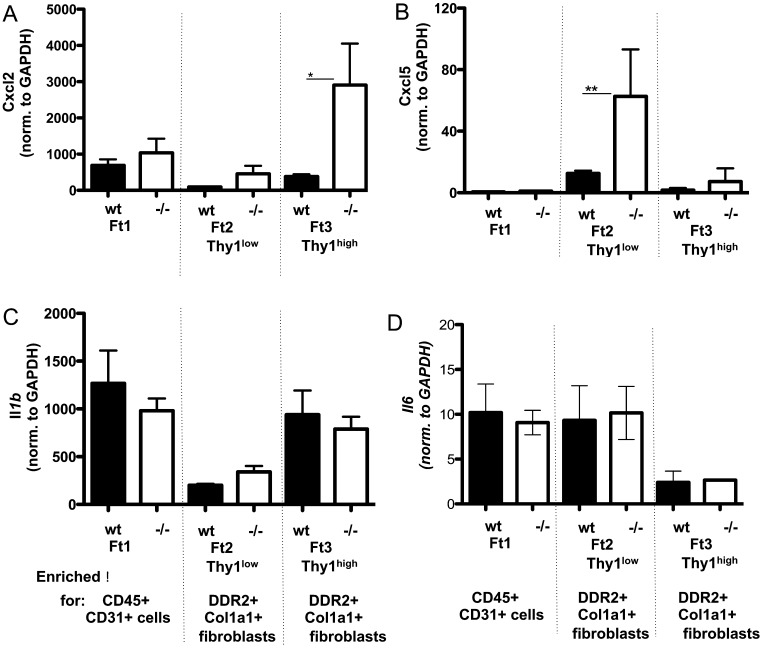
Cardiac fibroblasts express *Cxcl2* and *Cxcl5* post MI, which increase in the absence of intracellular glucocorticoid regeneration. qPCR analysis of *Cxcl2* (A) and *Cxcl5* (B), IL1b (C) and IL-6 (D) performed on RNA isolated from the cellular fractions 1–3 (Supplementary methods) in WT vs *Hsd11b1^−^^/^^−^* mice. *P < 0.05, **P < 0.01, *n* = 5–6 per group.

Cardiac fibroblasts express 11β-HSD1 (Furtado *et al*. 2014) and as ‘host’ cells in bone marrow chimera mice, it was reasoned that fibroblasts were a potential site for regulation of neutrophil chemoattractant expression in the infarcted heart. Three cellular fractions were isolated by magnetic bead separation (see ‘Materials and methods’ section and Supplementary Fig. 4) from the infarct and infarct border of hearts from WT and *Hsd11b1^−^^/^^−^* mice collected 24 h post MI. *Cxcl2* was found to be preferentially expressed in the *Ddr2*+*Col1a1+Thy1*^high^ fibroblast fraction from WT hearts (Fraction 3; Supplementary Fig. 5A; *P* < 0.01), and expression was increased 10-fold in this cellular fraction when isolated from hearts of *Hsd11b1^−^^/^^−^* mice (Fraction 3; Fig. 4A; *P* < 0.05). *Cxcl5* was preferentially expressed by *Ddr2*+*Col1a1+Thy1*^low^ fibroblast fraction (Fraction 2; Supplementary Fig. 5B; *P* < 0.005) and expression was significantly increased in fibroblasts isolated from hearts of *Hsd11b1^−^^/^^−^* mice relative to WT after MI (Fig. 4B; *P* < 0.01). *Hsd11b1* was expressed in both fibroblast subsets following MI (Supplementary Fig. 6). *Il-1* and *Il-6* were expressed in Fraction 1 containing CD45+ leucocytes and CD31+ve endothelial cells, as well as in fibroblasts, but expression levels did not differ between WT and *Hsd11b1^−^^/^^−^* animals in any fraction (Fig. 4C and D).

### 11β-HSD1 regulates the expression of *Cxcl2* and *Cxcl5* by cardiac fibroblasts *in vitro*

**Figure 5:**
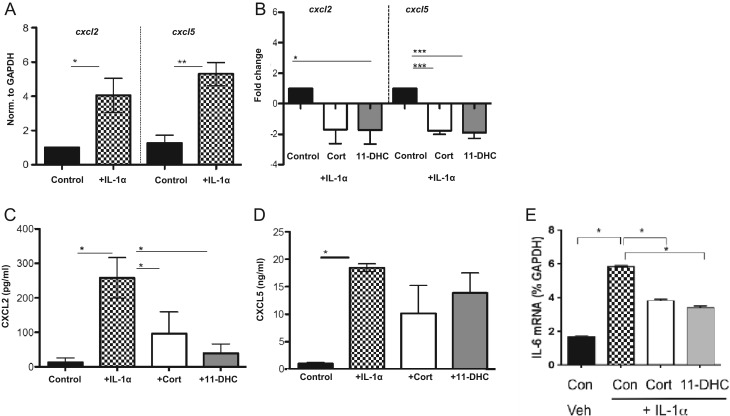
Cultured cardiac fibroblast expression of *Hsd11b1*, *Cxcl2*, *Cxcl5 and Il-6* in the presence or absence of IL-1α ± Cort or DHC. (A) qPCR analysis of *Cxcl2* and *Cxcl5* in cardiac fibroblasts cultured in the presence or absence of IL-1α for 24 h. (B) qPCR carried out on cardiac fibroblasts in the presence or absence of IL-1α ± corticosterone (Cort) or 11-DHC, expressed as fold change over IL-1α treatment alone. Protein production of CXCL2 (C) and CXCL5 (D) in the supernatants as measured by ELISA. (E) The influence of IL-1α ± corticosterone (Cort) or 11-DHC on expression of *Il-6* by cultured mouse cardiac fibroblasts. *P < 0.05, **P < 0.01, ***P < 0.005. *n* = 3–5 per group.

To confirm a role for glucocorticoid regeneration in regulating chemokine expression in cardiac fibroblasts, fibroblasts were isolated from the hearts of WT mice, cultured *in vitro* and activated by exposure to IL-1α for 24 h (Fig. 5). IL-1 induced an increase in the expression of *Cxcl2* and *Cxcl5* genes in cardiac fibroblasts (Fig. 5A) that was accompanied by increased secretion of CXCL2 and CXCL5 protein into the culture medium bathing these cells (Fig. 5C and D). Administration of corticosterone (200 nM) or of 11-dehydrocorticosterone (11-DHC; 200 nM), the product and substrate of 11β-HSD1, respectively, suppressed IL-1α-induced gene expression of *Cxcl2* and *Cxcl5* (Fig. 5B) and tended to reduce protein release (Fig. 5C and D). Although expression of *Il6* was not modified in fibroblasts freshly isolated from the heart, it was significantly increased by IL-1α in cultured cells, and this was suppressed by corticosterone and 11-DHC (Fig. 5E).

## Discussion

Neutrophils are rapidly recruited to the heart following injury and are required for tissue repair (Frangogiannis 2012, Yan *et al*. 2013), but regulation is essential as excessive or prolonged recruitment can result in increased tissue damage and impaired repair (Jordan *et al*. 1999, Ma *et al*. 2013). The results from the present study identify suppression of myocardial CXCL2 and CXCL5 chemoattractant expression by 11β-HSD1 as a novel mechanism with potential for regulation of neutrophil recruitment to the injured myocardium following infarction. Furthermore, the data indicate that cardiac fibroblasts are a key site for glucocorticoid regeneration by 11β-HSD1 during acute inflammation following myocardial injury.

Glucocorticoids, released acutely from the adrenal glands after MI, not only have direct anti-inflammatory effects, but also promote intracellular glucocorticoid regeneration from inert metabolites that enter cells from the circulation, thus amplifying their effects specifically in cells that express 11β-HSD1 (Chapman *et al*. 2013*a*, Gray *et al*. 2017). In the present study, systemic glucocorticoid was increased to the same extent in mice with and without 11β-HSD1 after MI; yet, in agreement with our previous observation (McSweeney *et al*. 2010), neutrophil recruitment to the heart was increased in the absence of 11β-HSD1. 11β-HSD1 deficiency or pharmacological inhibition similarly enhances neutrophil recruitment in other models of sterile inflammation, including arthritic joints, and in sterile peritonitis (Coutinho *et al*. 2012, Chapman *et al*. 2013*b*). Thus, intracellular regeneration of glucocorticoid in cells that express 11β-HSD1 promotes mechanisms that restrain neutrophil recruitment during inflammation.

Neutrophils express *Hsd11b1* and expression is increased as they are recruited to sites of inflammation (Kardon *et al*. 2008, Coutinho *et al*. 2016). Alteration in the expression of neutrophil adhesion molecules, including L-selectin, was identified as a mechanism associated with increased neutrophil recruitment that accompanies pharmacological inhibition of 11β-HSD1 in peritonitis (Coutinho *et al*. 2016). Such a mechanism could be involved in the post-MI heart, although expression of *L-selectin* was unchanged in hearts from *Hsd11b1*^−/−^ mice relative to WT. Increased neutrophil content of myocardium post MI was matched by increased blood neutrophil numbers. Bone marrow neutrophils express 11β-HSD1 at a higher level during inflammation (Coutinho *et al*. 2016), and we considered the possibility that this could influence neutrophil egress from the bone marrow. Egress is regulated by the balance between the actions of release signals (e.g. CXCL1, CXCL5) interacting with neutrophil CXCR2 and pro-retention signals in the bone marrow, particularly CXCL12 interacting with CXCR4 (Eash *et al*. 2010). Flow cytometry revealed that CXCR2 on bone marrow neutrophils from *Hsd11b1*^−/−^ mice post MI was not changed relative to WT but that expression of CXCR4 was reduced. Glucocorticoids upregulate CXCR4 expression on eosinophils and T-lymphocytes (Wang *et al*. 1998, Nagase *et al*. 2000), and this result suggests that glucocorticoid regenerated through 11β-HSD1 could engage a similar mechanism in neutrophils. However, in bone marrow chimeric mice, blood neutrophil numbers were increased in mice with ‘host’ knockout of *Hsd11b1*, but not in mice with bone marrow cell knockout of 11β-HSD1. Therefore, although an influence of neutrophil 11β-HSD1 activity cannot be excluded, activity in ‘host’ cells seem to be more central to the promotion of neutrophil mobilization after MI. This could include the non-myeloid component of the bone marrow itself and, although 11β-HSD1 failed to influence the expression of the retention factor CXCL12 in the bone marrow stroma, this merits further investigation.

The heart releases a number of CXCR2 ligands in response to injury (Frangogiannis 2012) and myocardial cells that express 11β-HSD1 likely represent a key ‘host’ component associated with the promotion of neutrophil mobilization post MI. Investigation of neutrophil chemoattractant expression revealed that while a number of these molecules, including *Cxcl1/KC*, were expressed at a higher level in the heart after MI, only a subset, including *Cxcl2* and *Cxcl5*, *IL-1* and *Il-6* were further increased in hearts from *Hsd11b1^−^^/^^−^* mice. Transfer of bone marrow from *Hsd11b1^−^^/^^−^* mice replaced myeloid components of the myocardium without altering neutrophil mobilization, suggesting that any alteration in gene expression relevant to this outcome is in non-myeloid cells of the heart. These cells include cardiomyocytes (Mazancova *et al*. 2005), smooth muscle cells (Hatakeyama *et al*. 2001) and fibroblasts (Sun & Myatt 2003). 11β-HSD1 enzyme activity is not detectable in endothelial cells (Christy *et al*. 2003, Dover *et al*. 2007). Neither mobilization to blood nor recruitment to the heart were increased following MI in mice with targeted deletion of *Hsd11b1* in smooth muscle cells and cardiomyocytes, ruling out 11β-HSD1 in these cells as being key in the regulation of neutrophil recruitment. This is consistent with our previous observations that *Hsd11b1* deletion in these cells did not influence angiogenesis or the development of heart failure following MI (White *et al*. 2016).

**Figure 6:**
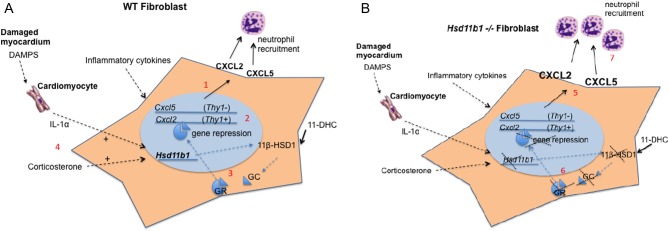
Schema for the regulation of neutrophil recruitment to the heart by 11β-HSD1. (A) Chemokines produced by fibroblasts in response to pro-inflammatory cytokines recruit neutrophils to the heart post MI (1). Thy1^high^ and Thy1^low^ cardiac fibroblasts preferentially produce CXCL2 and CXCL5, respectively (2). 11β-HSD1 catalyzes the regeneration of local glucocorticoid, dampening chemokine expression (3). Circulating corticosterone and IL-1α from necrotic cardiomyocytes increase 11β-HSD1 expression (4). (B) In the absence of 11β-HSD1, fibroblasts are driven to produce excess CXCL2 and CXCL5 (5), unleashed due to the lack of dampening local glucocorticoid (6), driving increased neutrophil recruitment to the infarcted heart (7).

Fibroblasts are abundant in the heart (Shinde & Frangogiannis 2014). They have immunomodulatory roles in many sites of the body (Smith *et al*. 1997, Silzle *et al*. 2004) regulating the types and functions of leukocytes recruited (Silzle *et al*. 2004). Cardiac fibroblasts secrete immunoactive molecules (Turner *et al*. 2011, Shinde & Frangogiannis 2014) and express 11β-HSD1 particularly highly (Furtado *et al*. 2014). In synovial fibroblasts 11β-HSD1 activity results in the suppression of inflammation associated with arthritis (Hardy *et al*. 2006, Hardy *et al*. 2008, Ahasan *et al*. 2012). To investigate whether 11β-HSD might have this role in the heart, *Ddr2+Col1a1+* fibroblasts were isolated from the mouse heart post MI. Cardiac fibroblasts expressed varying levels of *Thy1* and, interestingly, *Cxcl2* expression was specifically increased in *Thy1*^high^ fractions, whereas *Cxcl5* expression was increased in the *Thy1*^low^ fibroblast-enriched fraction. *Hsd11b1* expression was confirmed in both fractions (Fig. 6). Pro-inflammatory cytokines downregulate fibroblast *Thy1* expression (Hagood *et al*. 2005), and differences in cell expression may indicate subsets that have been exposed to different levels of cytokine or are in different states of activation in the healing infarct. Like *Cxcl2* and *Cxcl5*, *Il1b* and *Il6* expression was increased more in hearts from *Hsd11b1*^−/−^ mice after MI. Cultured human cardiac fibroblasts produce IL-1 and IL-6 in culture (Turner *et al*. 2007) and 11β-HSD1 was able to regulate the expression of *Il6* in cultured mouse cardiac fibroblasts in this study, as it does in cultured fibroblasts from other tissues (Hardy *et al*. 2006). However, there was no evidence for specific regulation of *Il1* or *Il6* expression by 11β-HSD1 in fibroblasts freshly isolated from the infarcted heart. This could reflect differences in the environment and in fibroblast phenotype *in situ* in the heart 24 h after induction of MI, compared to culture conditions. In fact, expression of *Il1b* and *Il6* in the cell fraction containing CD45+ve cells suggests that increased representation of mRNA from the increased number of recruited inflammatory cells in *Hsd11b1*^−/−^ relative to WT hearts is more likely to account for their higher expression in intact hearts from *Hsd11b1*^−/−^ mice.

IL-1α is induced early after ischemic injury in the heart, particularly by necrotic cardiomyocytes (Fig. 6) (Lugrin *et al*. 2015), and IL-1 receptor signaling is hypothesized to be critically important in regulating inflammatory pathways in the healing infarct, including promotion of pro-inflammatory cytokine release (Turner 2014). In cultured murine cardiac fibroblasts stimulated with IL-1α, provision of either corticosterone or 11-dehydrocorticosterone, the substrate for 11β-HSD1, decreased *Cxcl2* and *Cxcl5* expression, consistent with the regulation of gene expression secondary to 11β-HSD1 activity in these cells (Fig. 6). *Cxcl2* expression is known to be repressed by GR activation (Uhlenhaut *et al*. 2013), and *Cxcl5* was first described in mouse fibroblasts as a glucocorticoid-attenuated response gene (GARG) (Smith & Herschman 1996). Importantly, dampening of CXCL2 (Montecucco *et al*. 2013) and of CXCL5 (Chandrasekar *et al*. 2001) is known to reduce neutrophil-mediated tissue injury post MI. In future, it may be of value to further investigate the specific role of stromal cells in regulating neutrophil recruitment via these mediators, for example using an *in vitro* co-culture system (Munir *et al*. 2016).

Suppression of neutrophil recruitment is likely to be beneficial in terms of limiting acute injury to the myocardium, particularly following reperfusion. However, neutrophils secrete gelatinase-associated lipocalin that promotes macrophage polarization toward a pro-repair phenotype (Chen *et al*. 2014, Horckmans *et al*. 2017) and acquisition of this phenotype is also enhanced by efferocytosis of apoptotic neutrophils (Chen *et al*. 2014). Macrophage polarization toward a reparative ‘M2’ phenotype is enhanced in 11βHSD1-deficient mice during wound healing (McSweeney *et al*. 2010), and this associates with improved long-term functional outcome (White *et al*. 2016). Thus, early promotion of neutrophil recruitment to the myocardium may contribute to enhancement of tissue repair when 11β-HSD1 activity is lost. Alternatively, changes in the fibroblasts secretome under the influence of 11β-HSD1 may directly influence macrophage polarization status. While these data are supportive of cardiac fibroblasts as a key site for regulation of acute inflammation following myocardial injury, this mechanism needs to be more thoroughly tested in mice with fibroblast specific *Hsd11b1* deletion. Targeting *Cre-Lox*-mediated gene deletion specifically to fibroblasts has proved difficult in the past due to the lack of sufficiently selective *Cre−* mouse lines. However, *Col1a2 Cre* has recently been applied successfully to target fibroblasts during myocardial infarct healing (Duan *et al*. 2012), and in future, this approach will provide a means for testing the importance of *Hsd11b1* in fibroblasts, and other mesenchymal cells, post MI.

In conclusion, these data are consistent with a novel role for 11β-HSD1 in the regulation of acute inflammation following MI, via suppression of CXCL2 and CXCl5 chemoattractant expression and are supportive of cardiac fibroblasts as a key site for glucocorticoid regeneration by 11β-HSD1 following myocardial injury.

## Supplementary Material

Supporting Figure 1

Supporting Figure 2

Supporting Figure 3

Supporting Figure 4

Supporting Figure 5

Supporting Figure 6

## Declaration of interest

K J M, N A T, S A B, C J K, C I W, K M, R K, R S, V K, K E P, K E C and G A G have nothing to declare. B R W is an inventor on relevant patents owned by the University of Edinburgh and licensed to Actinogen Medical.

## Funding

This work was supported by the Wellcome Trust (Wbib91720MA and Wbib83184) and British Heart Foundation 4-year PhD studentship to C W (FS/09/053). The Centre for Cardiovascular Science is supported by a British Heart Foundation Centre of Research Excellence Award. S A B is supported by British Heart Foundation project grant (PG/11/110/29248) awarded to N A T and K E P.

## Author contribution statement

G A G and K J M conceived, designed the experiments and wrote the manuscript. K J M carried out the experiments and analyzed the data. N A T, S A B, C J K, C I W, K M, R A K, R S, V K and K E P contributed to the experiments. B R W and K E C reviewed the manuscript.
