# Supplementary material for: 11β-HSD1 suppresses cardiac fibroblast CXCL2, CXCL5 and neutrophil recruitment to the heart post MI
Source: J Endocrinol. 2017 Apr 11;233(3):315–27. doi: 10.1530/JOE-16-0501 (PMC5457506; doi:10.1530/JOE-16-0501)
Supplement: Supporting Figure 1 [file joe-233-315-s001.pdf]

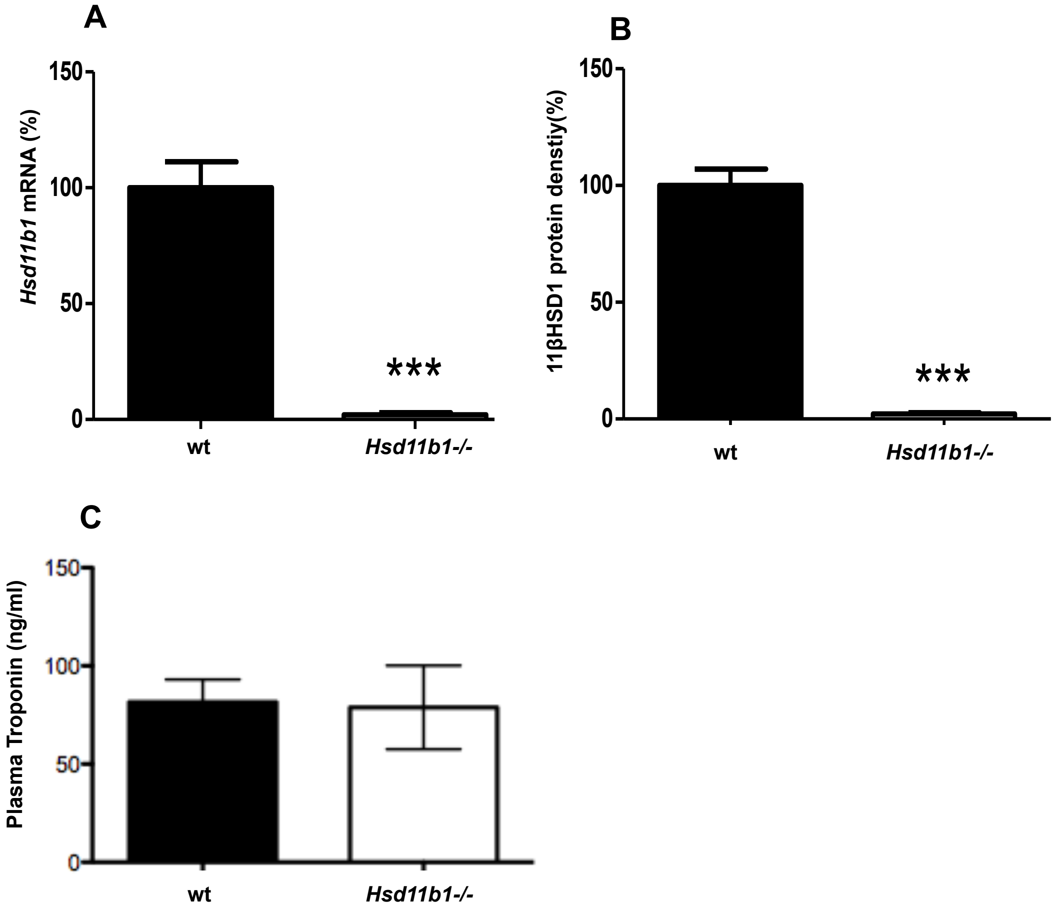

Supplementary Figure 1. qPCR (A) and western blot (B) analyses of cardiac tissue revealed a complete absence of 11βHSD1 enzyme expression in HSD1 null mice. *** P<0.005 n=4/4 (C) Levels of troponin were measured in the plasma of mice 1 day post-MI. n=5/5
