# Supplementary material for: 11β-HSD1 suppresses cardiac fibroblast CXCL2, CXCL5 and neutrophil recruitment to the heart post MI
Source: J Endocrinol. 2017 Apr 11;233(3):315–27. doi: 10.1530/JOE-16-0501 (PMC5457506; doi:10.1530/JOE-16-0501)
Supplement: Supporting Figure 2 [file joe-233-315-s002.pdf]

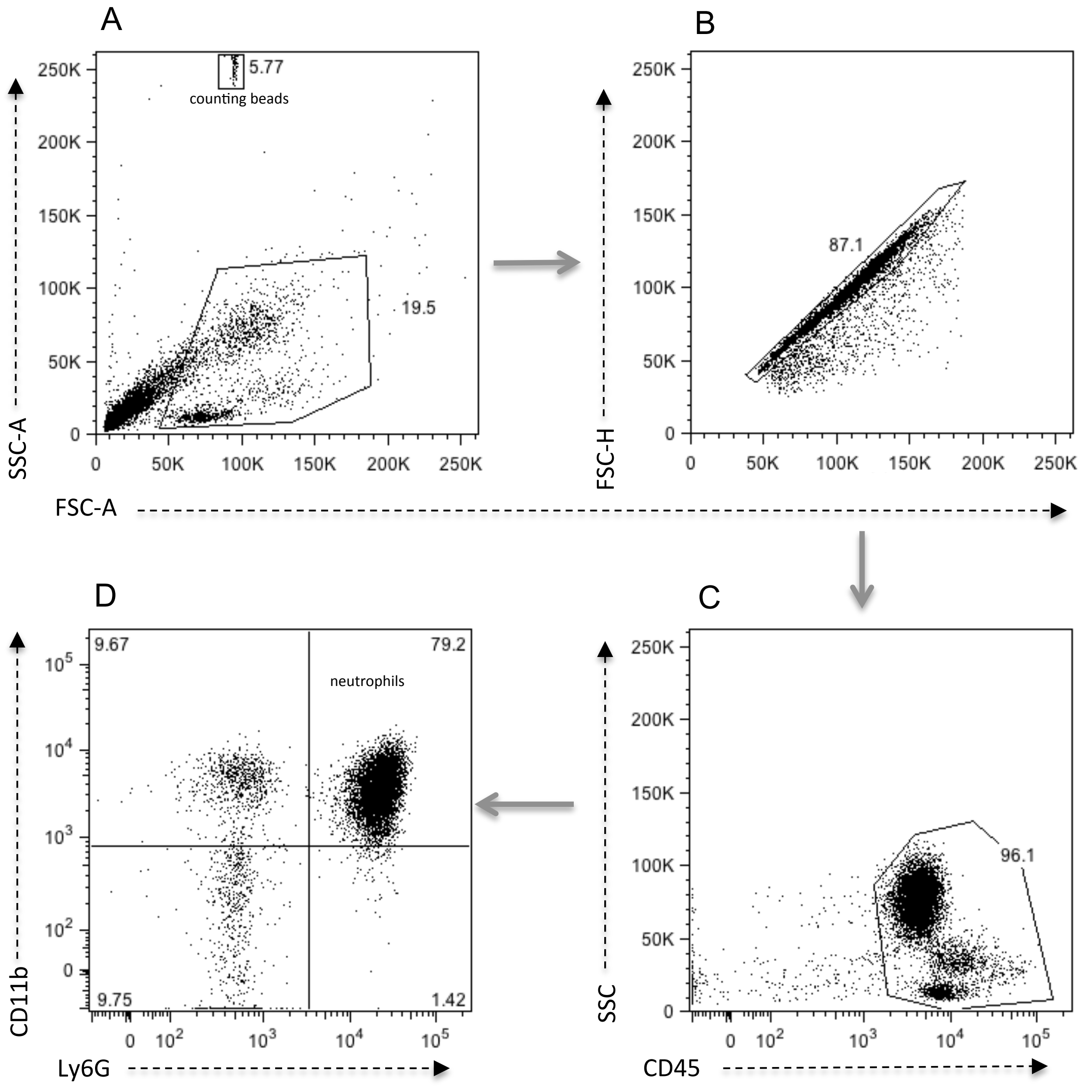

Supplementary Figure 2 . Gating strategy for flow cytometry analysis of blood neutrophils. Blood cells are gated by granularity and size (by SSC- Side Scatter and FSC, forward scatter) with counting beads added to ascertain absolute cell numbers (A). Singlets are selected to exclude cell clumps (B; FSC- H/FSC-A; -height/-area). CD45+ cells are selected (C) and of these, neutrophils are the CD11b + Ly6G+ cells (D).
