# Supplementary material for: 11β-HSD1 suppresses cardiac fibroblast CXCL2, CXCL5 and neutrophil recruitment to the heart post MI
Source: J Endocrinol. 2017 Apr 11;233(3):315–27. doi: 10.1530/JOE-16-0501 (PMC5457506; doi:10.1530/JOE-16-0501)
Supplement: Supporting Figure 3 [file joe-233-315-s003.pdf]

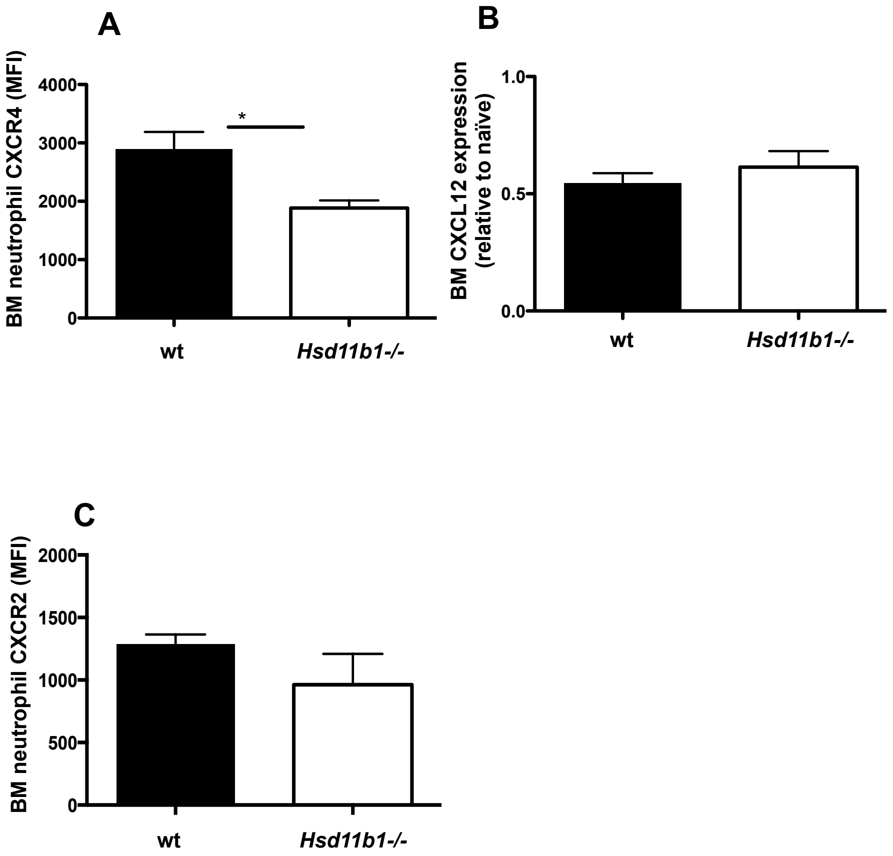

Supplementary Figure 3. Chemokine receptor protein expression in neutrophils was measured by flow cytometry (gating strategy defined in Supplementary Figure 2). CXCR4 expression, as calculated by mean fluorescence intensity (MFI) value, was lower in bone marrow of *Hsd11b1-/-* neutrophils 1 day post-MI than WT (A; $P<0.05$). CXCR2 expression was similar on bone marrow neutrophils between the two groups (C). Expression of the retention factor *Cxcl12* in the bone marrow of WT and *Hsd11b1-/-* animals post-MI was measured by qPCR (B; n=4/3).
