# Supplementary material for: 11β-HSD1 suppresses cardiac fibroblast CXCL2, CXCL5 and neutrophil recruitment to the heart post MI
Source: J Endocrinol. 2017 Apr 11;233(3):315–27. doi: 10.1530/JOE-16-0501 (PMC5457506; doi:10.1530/JOE-16-0501)
Supplement: Supporting Figure 4 [file joe-233-315-s004.pdf]

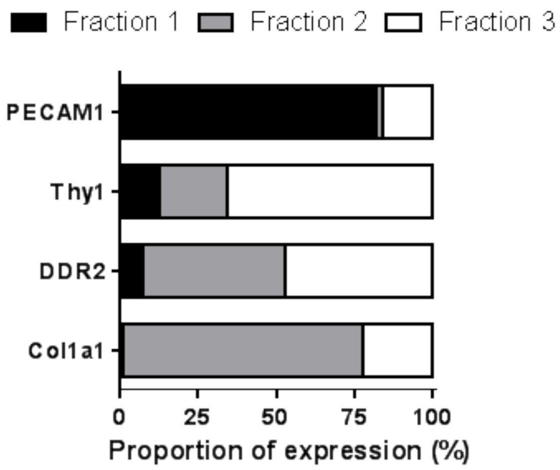

Supplementary Figure 4. Characterisation of cell types isolated from fibroblast enrichment. qPCR analysis of RNA from isolated fractions 1-3.
