# Supplementary material for: 11β-HSD1 suppresses cardiac fibroblast CXCL2, CXCL5 and neutrophil recruitment to the heart post MI
Source: J Endocrinol. 2017 Apr 11;233(3):315–27. doi: 10.1530/JOE-16-0501 (PMC5457506; doi:10.1530/JOE-16-0501)
Supplement: Supporting Figure 5 [file joe-233-315-s005.pdf]

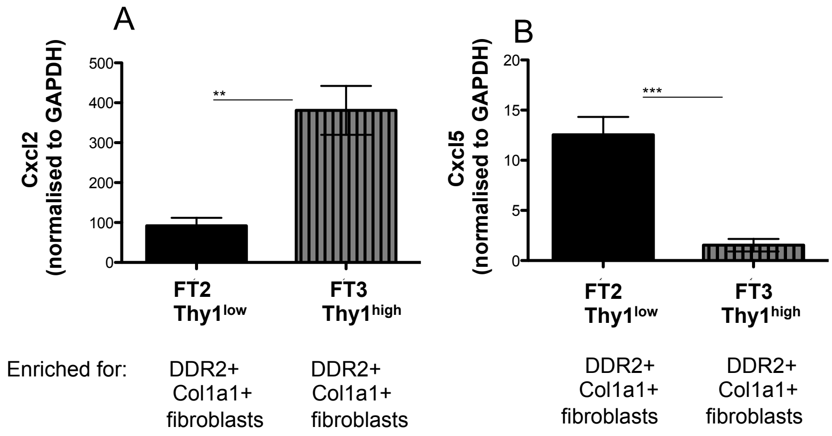

Supplementary Figure 5. To determine the response in cardiac fibroblasts under "normal" conditions qPCR analysis of *Cxcl2* (A) and *Cxcl5* (B) was performed on RNA isolated from the cellular fractions 2 and 3 (Ft2/3) in *wt* mice. Values were relative to whole infarct tissue and normalised to GAPDH **p<0.01, ***P<0.005, n=5-6 per group.
