# Supplementary material for: 11β-HSD1 suppresses cardiac fibroblast CXCL2, CXCL5 and neutrophil recruitment to the heart post MI
Source: J Endocrinol. 2017 Apr 11;233(3):315–27. doi: 10.1530/JOE-16-0501 (PMC5457506; doi:10.1530/JOE-16-0501)
Supplement: Supporting Figure 6 [file joe-233-315-s006.pdf]

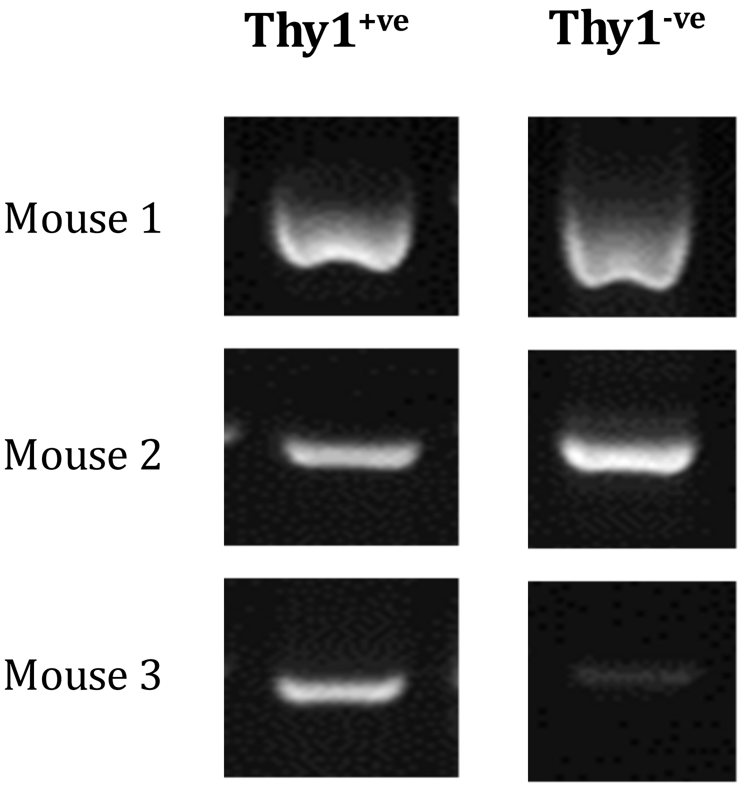


**Supplementary Figure 6:** *Hsd11b1* gene expression is detected by PCR in Thy1$^{+ve}$ and Thy1$^{-ve}$ fractions of the DDR$^{+ve}$, Col1a1$^{+ve}$ mouse cardiac fibroblast population, isolated from 3 separate mouse hearts, 24h after induction of myocardial infarction by coronary artery ligation. The bands represent a 587bp product corresponding to murine *Hsd11b1*. Primers were Forward:
5’-GTCCCTGTTTGATGGCAG-3, and
Reverse: 5’-AGGATCCAAGCAAACTTGCTTGCA-3.
